# Supplementary material for: Biodiversity data obsolescence and land uses changes
Source: PeerJ. 2016 Dec 13;4:e2743. doi: 10.7717/peerj.2743 (PMC5157196; doi:10.7717/peerj.2743)
Supplement: Document S1 [file peerj-04-2743-s002.docx]

Article S2

**Standardization of land uses maps**

In order to allow estimation of land use changes between years, all maps were standardized according to (1) the type of land use and (2) how information within each polygon was made available.

Maps dated 2000 and 2012 were comparable as both shared the same land use (LU) categories, whereas LU categories of maps dated 1956 and 1985 were different. We mapped all original LU categories to five new, simplified categories: forest (F), meadows/shrubland (MS), herbaceous crops (HC), woody crops (WC) and unproductive areas (UA) as follows:

Next, we assessed how the information was given in each map. All of them were polygon-based layers. For the 1956, 2000 and 2012 maps each polygon was attributed to one single LU category. On the contrary, each polygon in the 1985 layer was attributed a mixture of LUs. For example, one polygon could be characterized as having 80% of LU type 1 and 20% of LU type 2. In order to compare all four maps, we projected the LU data into a matrix, creating five LU attributes corresponding to the simplified LU categories (F, M, HC, WC and UA) for each polygon. Maps having originally single-LU polygons (1956, 200 and 2012 maps) would have 1 for the polygon’s new, simplified LU attribute and zero for the remaining four, while maps having multiple-LU polygons (1985 map) would reflect that proportion in the new attributes. This procedure allowed us to compare maps and track the LU changes between years.

**Establishment of the 25% threshold for land use changes**

The 25% threshold value was established in a pilot study performed on the cells that had a comparable sampling effort in two different periods. Twenty one cells met this requirement.

For every cell, the Morisita-Horn similarity index was calculated among the communities of each period. The index was related to the percentage of land use changes for the same period of time (Figure below).


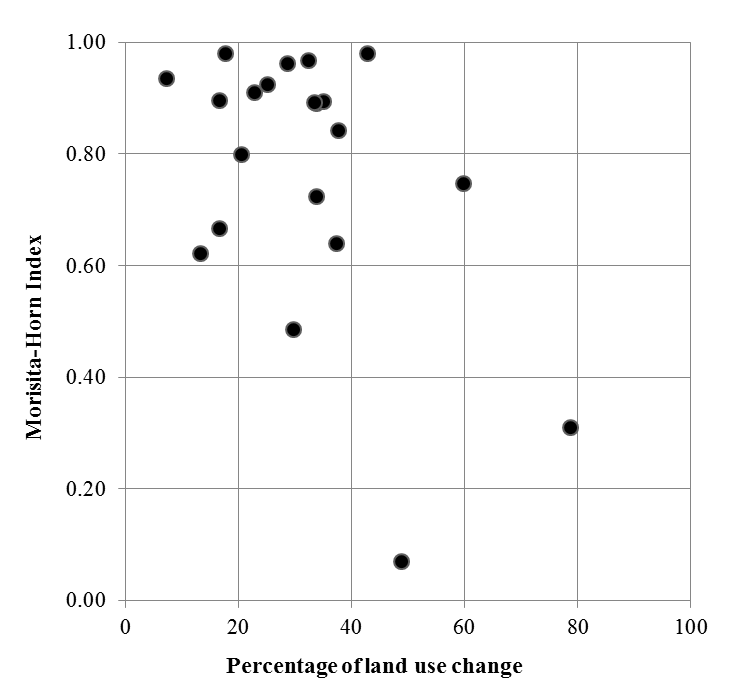


Percentage of land uses change and similarity are negatively related ( ). Small mammals’ communities remain similar over low percentages of land uses changes while more dissimilar communities are found in cells more disturbed. Then, we established a threshold of 25% of land uses changes to label cells as highly modified. We considered that this threshold allowed us to discriminate those cells where small mammals’ communities might have changed due to such land uses changes.
